# Relative Contribution of Prolyl Hydroxylase-Dependent and -Independent Degradation of HIF-1alpha by Proteasomal Pathways in Cerebral Ischemia

**DOI:** 10.3389/fnins.2017.00239

**Published:** 2017-05-17

**Authors:** Yomna Badawi, Honglian Shi

**Affiliations:** ^1^Neuroscience Program, University of KansasLawrence, KS, USA; ^2^Department of Pharmacology and Toxicology, University of KansasLawrence, KS, USA

**Keywords:** oxidative stress, proteasome, stroke, neurons, HIF-1

## Abstract

Hypoxia inducible factor-1 (HIF-1) is a key regulator in hypoxia and can determine the fate of brain cells during ischemia. However, the mechanism of HIF-1 regulation is still not fully understood in ischemic brains. We tested a hypothesis that both the 26S and the 20S proteasomal pathways were involved in HIF-1α degradation under ischemic conditions. Using *in vitro* ischemic model (oxygen and glucose deprivation) and a mouse model of middle cerebral artery occlusion, we tested effects of inhibitors of proteasomes and prolyl hydroxylase (PHD) on HIF-1α stability and brain injury in cerebral ischemia. We observed that 30 and 60 min of oxygen-glucose deprivation significantly increased the 20S proteasomal activity. We demonstrated that proteasome inhibitors increased HIF-1α stabilization and cell viability and were more effective than PHD inhibitors in primary cultured cortical neurons exposed to oxygen and glucose deprivation. Furthermore, the administration of the proteasome inhibitor, epoxomicin, to mice resulted in smaller infarct size and brain edema than a PHD inhibitor. Our results indicate that 20S proteasomes are involved in HIF-1α degradation in ischemic neurons and that proteasomal inhibition provides more HIF-1α stabilization and neuroprotection than PHD inhibition in cerebral ischemia.

## Introduction

Hypoxia inducible factor 1 (HIF-1), a transcription factor, is considered to be the most critical factor involved in the cellular response to hypoxia. This is mainly due to its regulation of 1–2% of human genes that play important roles in cellular adaptation to low oxygen (Mazure et al., 2004). HIF-1 is a heterodimeric protein formed by a continuously expressed subunit HIF-1β and an oxygen regulated subunit HIF-1α (Wang et al., 1995a). Under normal oxygen levels, HIF-1α is degraded through the ubiquitin-dependent proteasomal (26S) pathway. In order for a protein to be targeted to the 26S proteasome, it requires a poly-ubiquitin tail that can be detected by the 19S for subsequent processing and unfolding. It is known that von Hippel-Lindau tumor suppressor (pVHL) possesses ubiquitin ligase E3 activity that attaches ubiquitin to HIF-1α protein. To be recognized by pVHL, HIF-1α needs to be hydroxylated by prolyl hydroxylases (PHD), which uses oxygen as a co-substrate and hydroxylate two proline residues (Pro402 and Pro564). During hypoxia, HIF-1α is stabilized in cells due to reduced hydroxylation. However, low oxygen does not guarantee an increase in the level of HIF-1α protein. For example, HIF-1α levels were very low in hypoxic neurons in the absence of glucose. The mechanism responsible for low expression of HIF-1α in these conditions remains unclear.

The proteasomal proteolytic pathways are the main mechanisms responsible for the degradation of abnormal or unwanted proteins, which contain 26S and 20S proteasomal pathways. The 26S proteasome is the most abundant form and is composed of a 20S catalytic core and two regulatory 19S caps (Coux et al., 1996). The 20S proteasome complex can exist on its own and unlike the 26S proteasome it does not target ubiquitinated proteins but may act on oxidized proteins (Coux et al., 1996). Although, both the 26S and 20S can degrade oxidized proteins, many studies have determined that the contribution of the 20S is significantly higher (Davies, 2001; Breusing and Grune, 2008; Jung and Grune, 2008). Since HIF-1α contains residues with redox properties such as cysteine and methionine, it is an oxidizable protein (Huang et al., 1996, 1998). This potentially makes HIF-1α a target for degradation by the 20S proteasome in an oxidizing environment. Indeed, Kong et al. reported that HIF-1α's turnover under hypoxia was possibly regulated by the ubiquitin-independent proteasomal (20S) degradation pathway (Kong et al., 2006, 2007).

Ischemia is characterized by an increase in reactive oxygen species (ROS) formation. We hypothesized that both the ubiquitin-dependent (26S) and the ubiquitin-independent (20S) proteasomal pathways contribute to HIF-1α degradation during ischemia. To test the hypothesis, we assessed effects of ischemia on the 26S and 20S proteasomal activities, evaluated the contribution of the proteasomal pathways to HIF-1α degradation during hypoxia, and determined the effect of HIF stabilization through proteasomal inhibition on neuronal viability and brain damage in *in vitro* and *in vivo* ischemia models. To our knowledge, this is the first study to examine the contributions of both 20S and 26S proteasomal pathways to HIF-1α degradation and neuronal injury during cerebral ischemia with *in vitro* and *in vivo* models. Our results demonstrated that the 20S proteasomal activity was increased by ischemia. Furthermore, the results confirmed a role of the 20S in HIF-1α degradation.

## Experimental procedures

### Culture of SH-SY5Y cells and primary cortical neurons

SH-SY5Y cells were cultured in Dulbecco's Modified Eagle's Medium (DMEM) with 10% fetal bovine serum (FBS) and antibiotics (penicillin-streptomycin 1:100) at 37°C in a humidified incubator gassed with 95% air and 5% CO_2_. Primary neurons were prepared from the cortical tissues of Sprague–Dawley rat brains at embryonic day 16 [E16] to E18 (Guo et al., 2008). Experiments were conducted 10–12 days following dissection. The University of Kansas Institutional Animal Care and Use Committee approved all procedures.

### Assessment of proteasomal activities

Proteasomal activity was measured as described previously (Fekete et al., 2005). Briefly, cells were washed with PBS (pH 7.4) and then lysed by 2 freeze-thaw cycles in a lysis buffer [25 mM HEPES (pH 7.8), 0.25 M sucrose, 10 mM MgCl_2_, 1 mM EDTA, and 1 mM dithiothreitol (DTT)]. The lysates were centrifuged at 11,000 RPM at 4°C for 30 min. Cell lysate proteins (10 μg) were incubated with 100 μL of proteasome activity assay buffer. The assay buffer for evaluation of 26S proteasome function consisted of 50 mM Tris (pH 7.4), 5 mM MgCl_2_, 2 mM DTT, 2 mM ATP, and the fluorogenic substrate Suc-LLVY-AMC (80 μM in 1% DMSO, Sigma-Aldrich). The buffer for determining 20S proteasome function contained 20 mM HEPES (pH 7.8), 0.5 mM EDTA, 0.03% SDS, and 80 μM Suc-LLVY-AMC. The assays monitor the hydrolysis of Suc-LLVY-AMC into AMC (7-amino-4-methly-coumarin), which is then detected with a fluorescence plate reader at ex 380 nm and em 440 nm.

### Biochemical protein degradation

Plasmid cDNA of HIF-1α was transfected to SY5Y cells. Cell extracts prepared in modified M2 buffer was incubated with anti-HIF-1α antibody and protein A-sepharose beads (Pharmacia) at 4°C overnight. The beads were precipitated and washed five times with M2 buffer. One tenth of the beads were used to verify the bound HIF-1α protein. Western blot for the proteasome 20S subunits (antibodies: Santa Cruz, 1:1,000) was included to confirm no contamination of the proteasomes. The protein on beads was incubated with H_2_O_2_ (0.03%) and Fe^2+^ (0.1 mM) in DMEM for 3 h. Precipitated HIF-1α protein with or without oxidation by H_2_O_2_ were incubated with 20S proteasome (Boston Biochem) to determine the proteasome's ability to degrade HIF-1α. To determine the ability of 26S proteasome, the precipitated HIF-1α was incubated with a cytosol fraction from SH-SY5Y cells and 26S proteasome (Boston Biochem). After HIF-1α was incubated with the proteasomes for 1 and 3 h, Western blotting was carried out to determine the level of HIF-1α. MG-132 was used to confirm that the degradation was in fact the result of proteasome activity.

### Ischemic models

#### In vitro

Oxygen-glucose deprivation (OGD) was used as an *in vitro* ischemia model, which mimics the loss of oxygen and glucose that occur in a stroke when blow flow is blocked. Cells were incubated in the absence of glucose with 1% O_2_ at 37°C in a humidified hypoxia chamber (Coy laboratory products). Neuronal viability was assessed using the MTT [3-(4,5-dimethylthiazol-2-yl) 2,5-diphenyl tetrazolium bromide] assay kit (Invitrogen). To inhibit proteasomes and PHD activity, cells were pre-treated for 60 min with the proteasome inhibitors MG-132 (10, 40, and 80 μM) and epoxomicin (Epox, 8 μM) from Boston Biochem. Prolyl hydroxylases were inhibited with 2 mM dimethyloxalylglycine (DMOG).

#### In vivo

Brain ischemia was induced using the well-established middle cerebral artery occlusion (MCAO) model in mice (Clark et al., 1997). The University of Kansas Institutional Animal Care and Use Committee approved all *in vivo* brain ischemia studies (protocol #191). Anesthesia for the mice was induced with 3% isoflurane and maintained with 1.5% isoflurane throughout the procedure. Buprenorphine was used as the analgesic and was injected pre-operatively at 0.05 mg/kg. Twenty to twenty-five grams C57/Bl/6 male mice were subjected to MCAO followed by a 24 h period of reperfusion. To inhibit proteasomes and PHD activity, DMOG (50 mg/kg/0.1 cc, i.p.) and/or Epox (1.1 mg/kg/0.1 cc, i.p.) in DMSO were administered to mice 24 h before MCAO. TTC (2,3,5-triphenyltetrazolium chloride monohydrate) staining was used to assess brain damage (Ito et al., 1997). The corrected infarct area was calculated as previously described (Schäbitz et al., 1999). Brain edema volume (V_edema_) was also measured from the coronal sections that were stained by TTC by determining the volumes of both the ipsilateral (affected) hemisphere (V_Ipsi_) and the contralateral hemisphere (V_contra_) and using the equation: V_edema_ = V_contra_ − V_Ipsi_ (Yan et al., 2011). The final analyses of the different treatments and animal groups were performed in a blinded fashion.

### Immunoblot analysis

Cells were lysed in 200 μL RIPA buffer (ThermoScientific) and the protease inhibitor cocktail kit (Thermo Scientific) and scraped using a cell lifter (Biologix Research Company). The lysates were centrifuged at 12,000 RPM for 10 min at 4°C and the protein concentration of the supernatants was determined using a protein assay kit (Bio-Rad). Proteins were separated by SDS–PAGE and the separated proteins were transferred to a nitrocellulose membrane (BIO-RAD). After being blocked with 5% nonfat milk in Tris-buffered Saline with Tween (TBST the membrane was incubated with the anti-HIF-1α rabbit monoclonal (1–1,000; 04–1,006; Millipore) or the anti-hydroxyl-HIF rabbit polyclonal (1–1,000; NB110-74679 Novus) primary antibody overnight at 4°C and the secondary antibody (1–3,000; goat anti rabbit IgG-horseradish peroxidase; sc-2030 Santa Cruz) for 1 h at RT. Western blots were quantified using ImageJ software and protein levels were normalized to β-actin.

### Data and statistical analysis

Data are presented as means ± SEM from a minimum of three independent experiments. One-way ANOVA followed by Tukey's multiple comparisons test and the Student's *t*-test were used for overall significance. Differences of *p* < 0.05 were considered statistically significant. GraphPad Prism software, Image-Pro Plus 5.1 (Media Cybernetics), ImageJ, and Excel were used for data analyses.

## Results

### *In vitro* ischemia elevated proteasomal activity in neurons

We first evaluated the effects of ischemia on proteasomal activity in neurons. Primary cortical neurons were exposed to OGD for 30, 60, and 90 min and then the activities of the 26S and 20S proteasome were assessed and compared to neurons at control conditions. Following OGD treatment, there was a mild increase in 26S activity however it was not statistically significant (Table 1). On the other hand, 20S activity was significantly increased after 30 or 60 min exposure to OGD. The activity began to decrease after 60 min. Since ischemia is characterized by an increase in ROS (Moro et al., 2005) we also determined the effect of KO_2_ generating superoxide anion radical and H_2_O_2_ on proteasomal activity in SH-SY5Y cells (Supplementary Figure 1). Our results indicated that ROS can also increase both 20S and 26S activities dependent on its concentration.

**Table 1:** **Effect of oxygen-glucose deprivation on 26S and 20S proteasomal activity in primary cortical neurons**.

**OGD duration (min)**	**Proteasomal activity (% of control)**	
	**26S**	**20S**
0	100	100
30	106.9 ± 5.5	104.8 ± 2.5^*^
60	107.1 ± 3.9	114.4 ± 4.1^*^
90	105.2 ± 2.8	100.5 ± 5.5

*Primary cortical neurons were exposed to oxygen and glucose deprivation (OGD) for 30, 60, or 90 min and then the 26S and 20S proteasomal activity was determined from the lysates. The increase in the 20S proteasome activity following both 30 and 60 min OGD exposure was found to be significant*.

* *p < 0.05 vs. 0 min (n = 3)*.

### 20S proteasomes were able to degrade HIF-1α in a biochemical system

**Figure 1:**
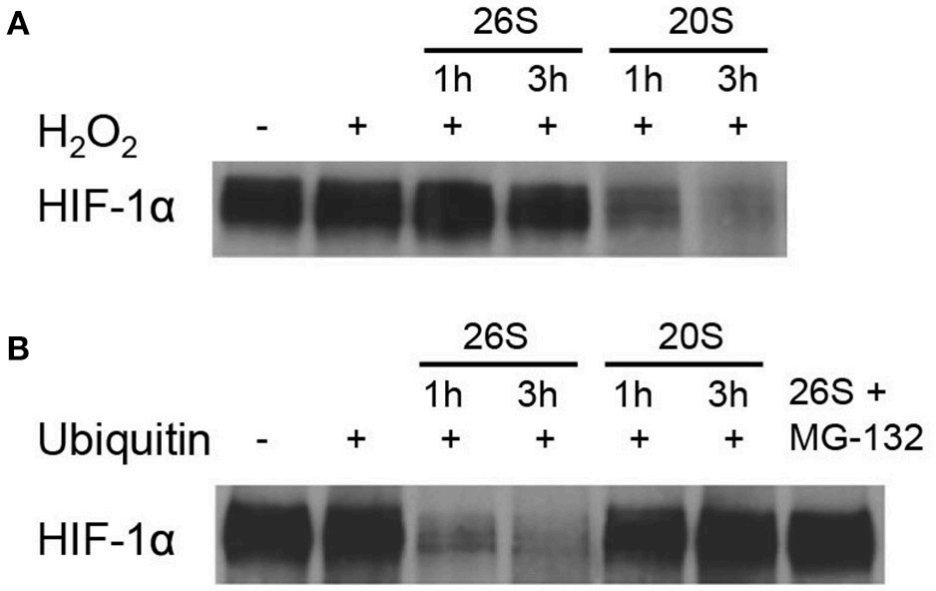
**HIF-1α degradation by the 20S proteasomes**. Immunoblotting for HIF-1α after the HIF-1α protein-beads were incubated with a cytosol fraction from SH-SY5Y cells and either the 26S or 20S proteasome for 1 and 3 h with the addition of **(A)** H_2_O_2_ or **(B)** ubiquitin.

We investigated the effect of 20S on the level of HIF-1α protein at elevated ROS conditions in a biochemical system. As shown in Figure 1, H_2_O_2_ treatment alone had no effect on the level of HIF-1α protein. 20S proteasomes efficiently degraded HIF-1α protein exposed to H_2_O_2_ for 1 or 3 h while 26 proteasomes had little effect on the level of HIF-1α. The figure also demonstrates that in the presence of ubiquitin, 26 proteasomes readily degraded native HIF-1α whereas 20S proteasomes had no effect on the level of HIF-1α. The results indicate that 20S and 26S proteasomes prefer degrading oxidized and native HIF-1α protein, respectively.

### Both 20S and 26S proteasomes contributed to HIF-1α protein degradation *In vitro*

**Figure 2:**
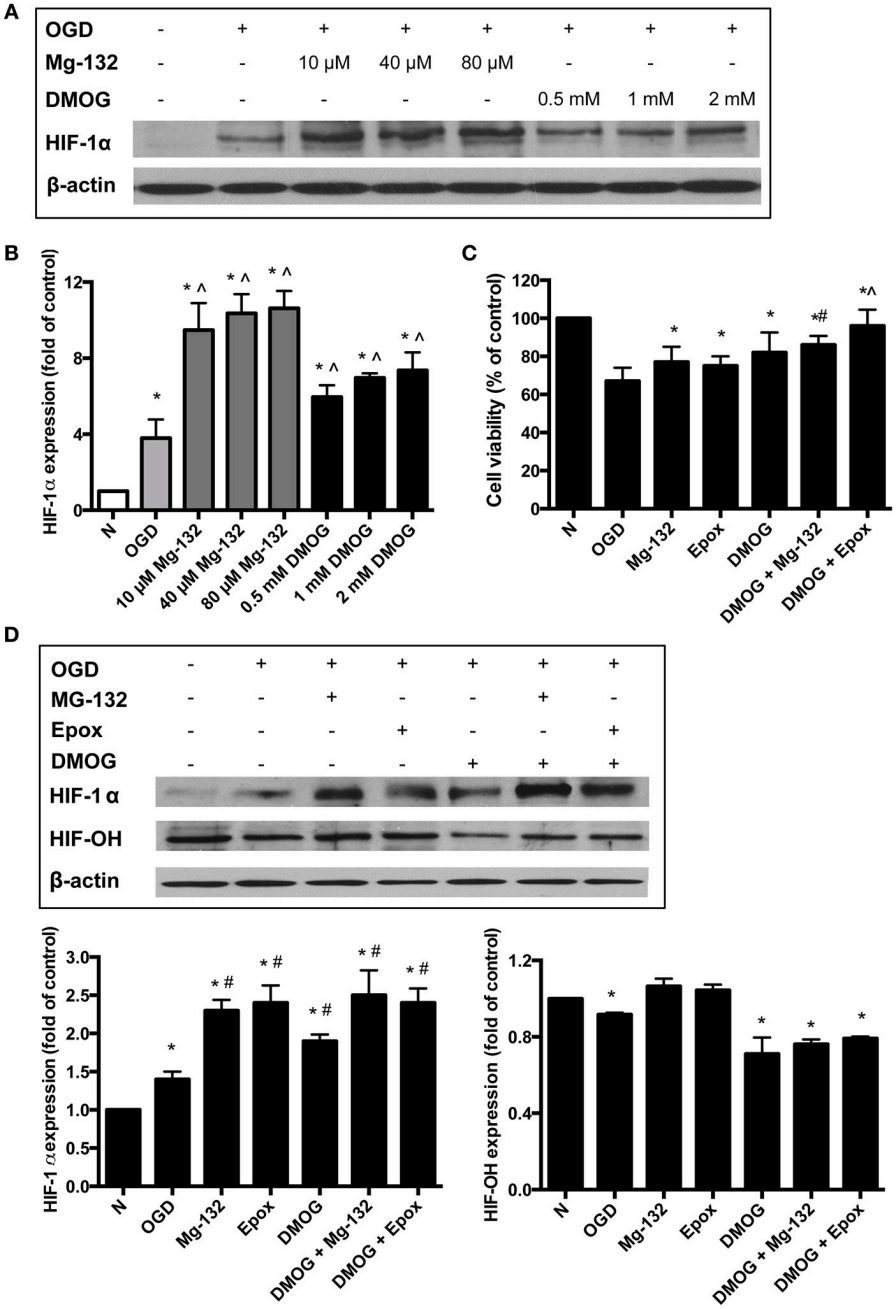
**HIF-1α protein stabilization with the treatments of proteasome or PHD inhibitors. (A)** Immunoblotting showing HIF-1α protein levels in neurons and **(B)** the quantitative results for Western blot data. Equalization of protein loading was determined using β-actin as the housekeeping protein. ^*^p < 0.05 vs. normoxia (N), ^∧^*p* < 0.05 vs. oxygen/glucose deprivation (OGD) (*n* = 3). **(C)** Neuronal viability assessed using the MTT assay ^*^p < 0.05 vs. OGD, ^#^*p* < 0.05 vs. DMOG (*n* = 3). **(D)** Immunoblotting showing HIF-1α and hydroxyl-HIF-1α (HIF-OH) protein levels in neurons. Equalization of protein loading was determined using β-actin as the housekeeping protein. Quantitative results for Western blot data. ^*^p < 0.05 vs. N, ^#^*p* < 0.05 vs. OGD (*n* = 3).

The above results suggested that both 20S and 26S proteasomes could degrade HIF-1α although they preferred different modified forms of the protein. To determine the contributions of both 20S and 26S proteasomal pathways to the degradation of HIF-1α in ischemic neurons, we carried out experiments in the presence of proteasome inhibitors or PHD inhibitors. As hydroxylation is a required step for HIF-1α to be degraded through the 26S degradation pathway but not the 20S pathway, PHD and proteasomal inhibitions would differentiate the individual contribution of the two pathways to HIF-1α degradation. MG-132 and DMOG were used to inhibit proteasomes and PHD, respectively. As Figure 2 shows, OGD for 60 min increased the HIF-1α level in primary cultured cortical neurons. Pretreatment of MG-132 at 10 μM further elevated HIF-1α level (149% increase, compared to OGD only). MG-132 at 40 and 80 μM induced a 172 and 174% increase in the level of HIF-1α over OGD only, respectively. A smaller increase in the HIF-1α level in the presence of higher level of MG-132 indicated that at a level of 10 μM MG -132 was able to effectively inhibit proteasomal activity.

DMOG was also able to further increase the HIF-1α level, indicating that even under low oxygen conditions, 26S proteasomal pathway was involved in degrading HIF-1α protein. We observed a 57% increase in the HIF-1α protein level in cells pretreated with 0.5 mM DMOG. At 1 and 2 mM, DMOG caused 83 and 93% increase, respectively, in the protein level. A higher concentration of DMOG did not cause further increase in the HIF-1α level (data not shown), indicating that inhibition of hydroxylation by DMOG at 2 mM is close to its maximal effect. Taken together, these data suggested that inhibiting the proteasomes with MG-132 stabilized the HIF-1α level more than DMOG did under the OGD condition.

Next we treated the neurons with a combination of both a proteasome inhibitor (40 μM MG-132 or 8 μM Epox) and the prolyl hydroxylase inhibitor, DMOG (2 mM). Neuronal viability was significantly increased with the combination treatment compared to the control normoxic conditions, OGD, and the individual drug treatments (Figure 2C).

Western blot analysis also showed that the combined drug treatments lead to a greater stabilization of HIF-1α (Figure 2D). Immunoblotting analysis for hydroxylated HIF-1α (HIF-OH) confirmed that DMOG reduced hydroxylation of HIF-1α. The level of HIF-OH was highest under normoxia. It was decreased under OGD exposure and slightly increased with the inhibition of the proteasomes. The combined drug treatment resulted in HIF-OH levels that were more than that of the DMOG treatment only but still lower than that with the proteasome inhibitors alone.

### Proteasomal inhibition is more effective than PHD inhibition in reducing brain infarct size in an *In vivo* stroke model

**Figure 3:**
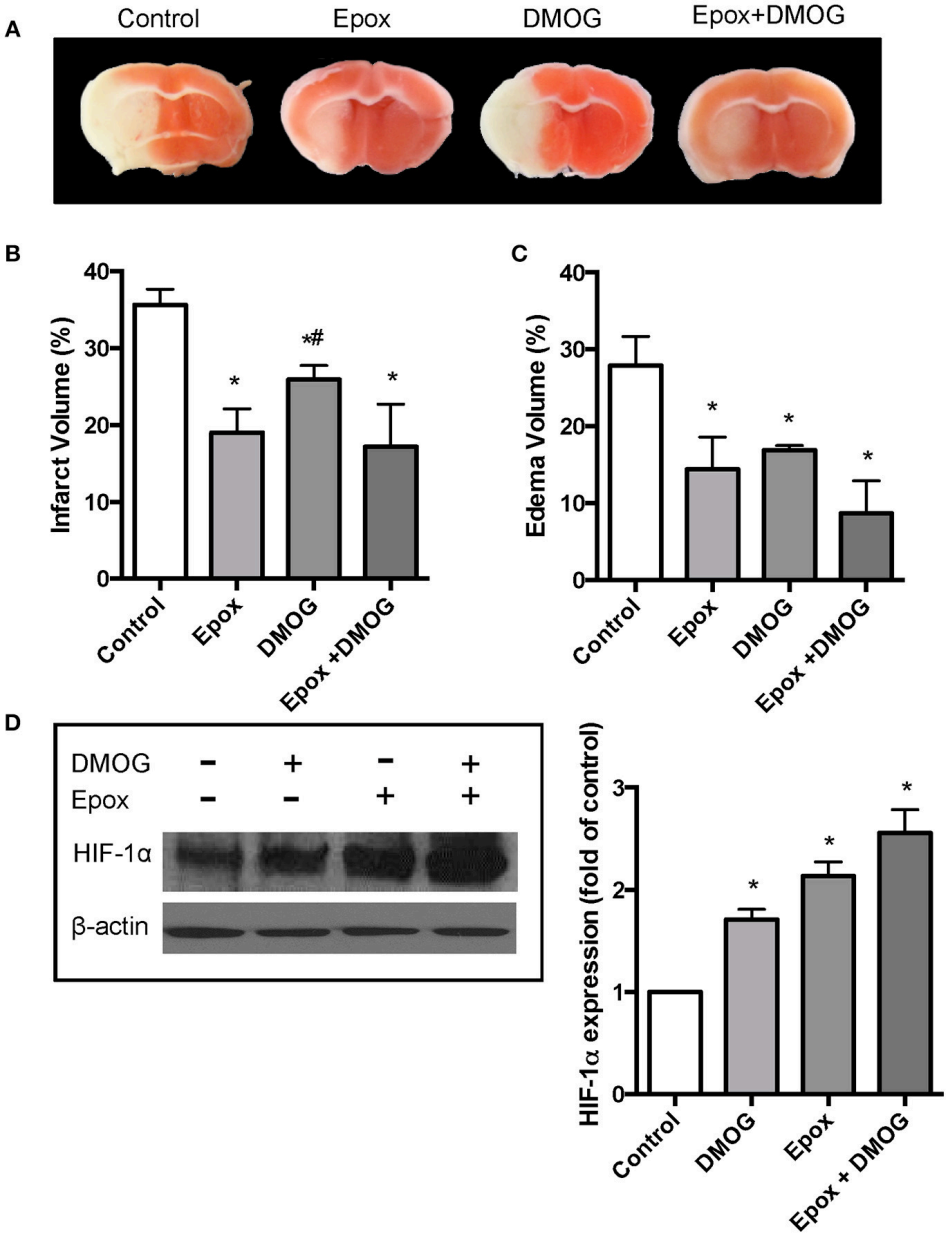
**Effect of DMOG and epoxomicin (Epox) on HIF-1α expression and brain damage induced by cerebral ischemia**. Brain damage as determined by TCC staining after mice were subjected to 90 min ischemia followed by 24 h reperfusion. **(A)** Representative TTC staining of brain coronal sections taken from the 3 mm position of the frontal pole. **(B)** Quantification of infarct volume determined by TTC stained sections (*n* = 5). Data presented as means ± SEM ^*^p < 0.05, vs. control untreated mice. ^#^*p* < 0.05, vs. mice treated with Epox. **(C)** Quantification of brain edema volume estimated from TTC stained sections (*n* = 5). Data presented as means ± SEM ^*^p < 0.05, vs. control untreated mice. **(D)** Immunoblotting showing the levels of HIF-1α from the ipsilateral brain hemispheres of untreated mice and mice treated with dimethyloxalylglycine (DMOG), Epox or a combination of both (*n* = 3).

We then evaluated effects of proteasomal inhibition and hydroxylase inhibition on brain damage in an *in vivo* mouse stroke model. Adult male mice were subjected to MCAO followed by a 24 h period of reperfusion. Four animal groups were examined: (1) Control, (2) Epox, (3) DMOG, and (4) Epox + DMOG. Epox was chosen as the proteasome inhibitor for the *in vivo* studies because it is able to cross the blood-brain barrier (Stefanis and Keller, 2007). It is also more selective and potent than MG-132. Epox was administered at 1.1 mg/kg/0.1 cc as reported previously (Meng et al., 1999). DMOG was administered at 50 mg/kg/0.1 cc to inhibit the PHD (Ogle et al., 2012). The results revealed that Epox was more effective at reducing infarct size compared to DMOG (Figure 3B). The brain images demonstrating the ischemic infarct from all analyzed animals are shown in Supplementary Figure 2. We also assessed the infarct size following MCAO with the pretreatment with higher concentrations of DMOG to ensure that a sufficient amount of DMOG was given to the mice to inhibit the PHD (Supplementary Figure 3). Epox was more effective than the increased concentrations of DMOG. Brain edema volume was also measured from the coronal sections. As shown in Figure 3C, all 3 drug-treated animal groups had a significant decrease in edema volume following MCAO compared to the control mice. However, there was no significant difference between the groups. Furthermore, immunoblotting showed that HIF-1α protein levels were increased in the ipsilateral hemisphere and that it is significantly stabilized with the combined administration of Epox and DMOG.

## Discussion

The induction of HIF-1 is very important following cerebral ischemia. Dimerization of HIF-1α to HIF-1β leads to the expression of various genes that can promote cellular adaptation to conditions of low oxygen. Its targets include genes that code for molecules that participate in vasomotor control, angiogenesis, erythropoiesis, cell proliferation, and energy metabolism (Semenza, 2003a,b; Sharp and Bernaudin, 2004). Each of these functions potentially contributes to the survival of neuronal cells at hypoxic conditions. Neuron-specific HIF-1 deficient mice showed increased brain damage following MCAO (Baranova et al., 2007). In this study, we investigated the mechanisms of HIF-1α degradation in ischemic neurons. The results for the first time demonstrated that both 20S and 26S proteasomal pathways were involved in HIF-1α degradation in ischemic neurons. The results provide important information for not only understanding the pathophysiology of cerebral ischemia but also designing potential strategies for stroke treatment.

Under normoxic conditions, HIF-1α is rapidly degraded via the ubiquitin-dependent proteasomal (26S) degradation pathway after hydroxylation and ubiquitination. Under hypoxic conditions, it is generally regarded that HIF-1α is accumulated due to hydroxylation inhibition (Jiang et al., 1996; Wood et al., 1996; Salceda and Caro, 1997; Huang et al., 1998; Kallio et al., 1999; Huang and Bunn, 2003). Our experimental results from PHD inhibition suggest that the 26S proteasomal pathway contributed to the degradation of HIF-1α in ischemia, at least in our experimental setting, indicating that PHD activity was not completed suppressed at the low oxygen conditions. Besides the 26S pathway, there are other pathways, such as 20S, that may cause HIF-1α degradation. For HIF-1α to be a target of the 20S proteasomes it would have to be oxidized as the 20S preferably degrades oxidized protein. HIF-1α was found to be an oxidizable protein soon after it was discovered (Wang et al., 1995b; Huang et al., 1996) due to its residues with redox properties such as cysteine and methionine. Oxidative modification of cellular proteins was reported to occur within 10 min and peak at 1 or 2 h after the ischemic insults (Oliver et al., 1990; Hall et al., 1995). Yet, it is not known which residue of HIF-1α is oxidized by ischemia. Our observation that proteasomal inhibition increased HIF-1α levels more than a PHD inhibitor could under a low O_2_ condition is in accordance with a previous report (Demidenko et al., 2005). These results indicate that HIF-1α is, at least partly, degraded via a hydroxylation-independent proteasome pathway, and suggest that besides 26S, the 20S proteasome is involved in the degradation of HIF-1α under hypoxia.

Ischemia induces a rapid accumulation of free iron the brain (Palmer et al., 1999). Given that PHDs belong to a family of enzymes that require iron as an essential cofactor, the increase in the iron level elevates PHD activity. Under iron-lacking conditions, PHDs are inactivated, preventing the ubiquitination and proteasomal degradation of HIF-1α. In this aspect, the neuroprotective effects of iron chelators such as desferoxamine have been attributed to the activation of HIF-1 (Prass et al., 2002; Hamrick et al., 2005; Freret et al., 2006; Sorond et al., 2015). Free iron plays a critical role in oxidative damage due to the Fenton's reaction where hydrogen peroxide is converted to the highly reactive hydroxyl radical. Based on our results, it is possible that iron chelators may also enhance HIF-1α expression by inhibiting its oxidation and thus its degradation through the 20S proteasomal pathway. Indeed, reduction of ROS by the liposoluble iron chelator 2,2′-dipyridyl in a rat photothrombotic ischemic stroke model was observed concomitantly with an increase in the level of HIF-1α protein (Demidenko et al., 2005).

It is noteworthy to point out that proteasomal inhibitors may protect the brain from ischemic injury by additional or alternative mechanisms. For example, it has been reported that the proteasome inhibitor MLN519 was neuroprotective against stroke injuries by inhibiting the pro-inflammatory nuclear factor-κB (Berti et al., 2003; Williams et al., 2003, 2004, 2005, 2006). Another proteasome inhibitor, bortezomib, was also able to reduce post-ischemic inflammation (Henninger et al., 2006; Zhang et al., 2010). Furthermore, a recent study by Doeppner et al. (2012) showed that the intracerebral delivery of BSc2118, a novel proteasome inhibitor, induced neuroprotection following ischemia by decreasing blood brain barrier breakdown and enhanced cell proliferation, neurogenesis and angiogenesis (Li et al., 2016). They speculated that the stimulation of angioneurogensis was most likely a consequence of HIF-1α stabilization. This is because in contrast to the intracerebral delivery of BSc2218, a systemic BSc2118 delivery did not affect HIF-1α abundance and subsequently there was no stimulation of angioneurogensis in the brain (Sorond et al., 2015). It is not clear if epoxomicin has additional functions besides inhibiting proteasomal activities. The application of proteasome inhibitors in this study was mainly to investigate the mechanism of ROS mediated HIF-1α degradation in neurons during ischemia. An additional increase in HIF-1α levels when both the proteasome and PHD are inhibited indicates a role of the 20S in HIF-1α degradation (Figure 2D). We also found that the combined treatment and the stabilization of HIF-1α were protective in both the *in vitro* primary neuron ischemia model and in the *in vivo* mouse stroke model in terms of maintaining cell viability (Figure 2C) and in the reduction of infarct size (Figure 3).

**Figure 4:**
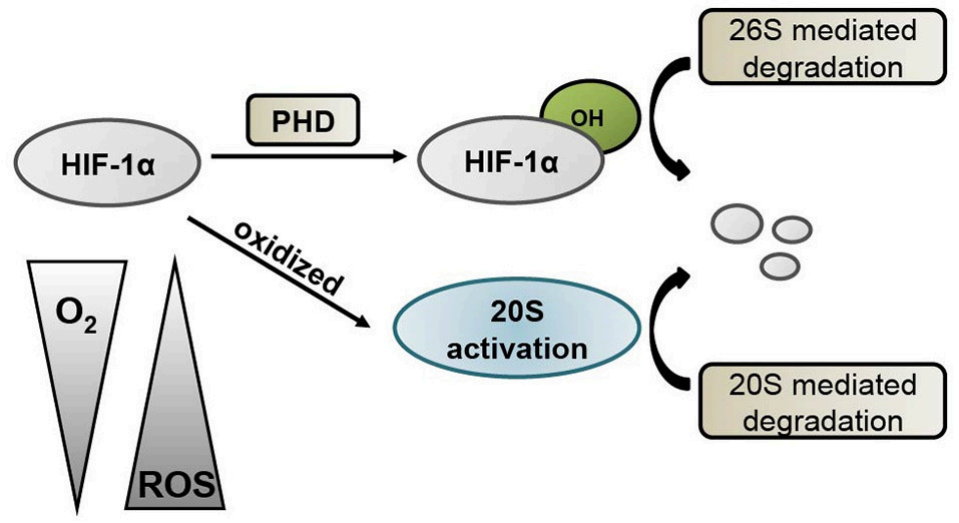
**Schematic diagram of HIF-1α degradation mechanisms**. Under normoxic conditions, HIF-1α is hydroxylated by prolyl hydroxylases (PHD), which targets it for degradation via the 26S proteasome pathway. During conditions of low oxygen and high oxidative stress, HIF-1α can be oxidized and subsequently degraded by the 20S proteasome.

Currently, PHD enzymes are being targeted for drug discovery in the treatment of stroke. PHDs regulate the HIF-1α degradation pathway by acting as oxygen sensors (Epstein et al., 2001). Therefore, the inhibition of PHD leads to the activation of HIF and its downstream genes. A recent report demonstrated that neuronal inactivation of PHD2 is sufficient to improve stroke recovery with improved histological and functional outcome by increasing HIF-1 activity (Li et al., 2016). PHD inhibitors have been shown to be protective when administered prior to or upon reperfusion in brain ischemia models (Gidday et al., 1994; Prass et al., 2002; Siddiq et al., 2005; Liu et al., 2009; Ogle et al., 2012). However, our results suggest that inhibiting PHD may only provide a partial effect because HIF-1α can be degraded by other pathways such as the 20S proteasomes (see Figure 2A). The results reported here reveal that not only the 26S proteasomal pathway but also the 20S one are involved in HIF-1α degradation (Figure 4). Thus, PHD inhibitors are not as effective as the proteasome inhibitors because the PHD inhibitors block hydroxylation of HIF-1α and the 26S pathway activity, but not the 20S pathway.

## Conclusions

In summary, HIF-1 plays an important role in the fate of ischemic neurons. Understanding the mechanism of HIF-1 induction is very important in determining its role in cerebral ischemia and providing potential approaches to regulate its expression. This proof-of-concept study demonstrate that ischemia can alter proteasomal activities in neurons and that both 26S and 20S proteasomal degradation pathways contribute to the HIF-1α degradation. ROS consist of several unique species, which have dramatically different reactivities and half-lives. Actions of ROS on HIF-1α degradation may be through specific ROS, rather than ROS in general. Understanding the different ROS species involved will provide us with a better understanding of the mechanisms. It will also help us design more effective intervention strategies, such as efficient antioxidants, to inhibit or remove the specific species. We also provide evidence that the inhibition of proteasomal activity with MG-132 and Epox restored the attenuated accumulation of HIF-1α that might result from increased ROS and provided neuroprotection. Regulating HIF-1α induction and the genes induced by HIF-1 under ischemia are highly promising therapeutic targets for cerebral ischemia (Giaccia et al., 2003; Williams et al., 2004; Shi, 2009). Defining this mechanism for HIF-1α degradation will make it possible to design more efficient agents for inhibiting HIF-1α degradation and promoting its neuroprotective properties.

## Author contributions

YB and HS designed the experiments. YB performed the experiments. YB and HS analyzed the data and wrote the manuscript.

### Conflict of interest statement

The authors declare that the research was conducted in the absence of any commercial or financial relationships that could be construed as a potential conflict of interest.
