# Supplementary figures and images for: Relative Contribution of Prolyl Hydroxylase-Dependent and -Independent Degradation of HIF-1alpha by Proteasomal Pathways in Cerebral Ischemia
Source: Front Neurosci. 2017 May 17;11:239. doi: 10.3389/fnins.2017.00239 (PMC5434458; doi:10.3389/fnins.2017.00239)

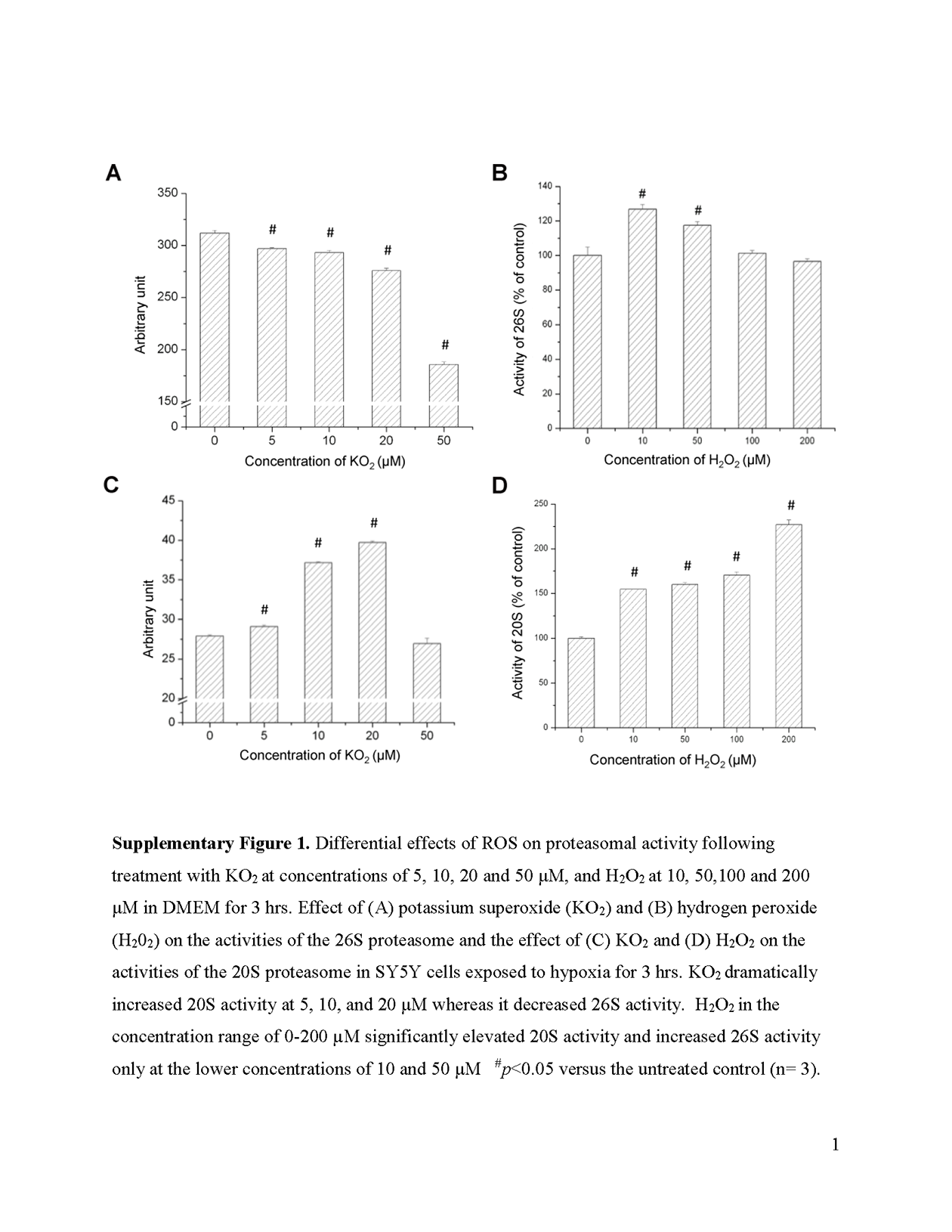

Supplement: Supplementary file 1 [file Image1.TIF]

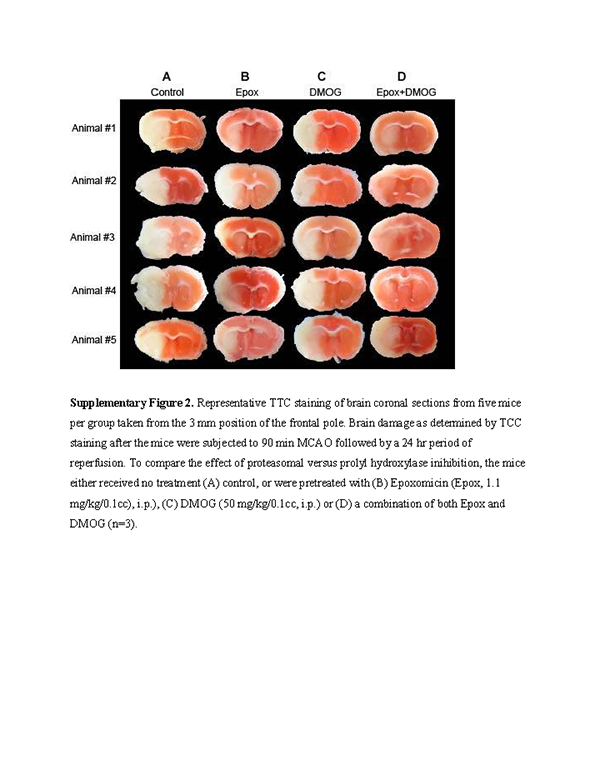

Supplement: Supplementary file 2 [file Image2.TIF]

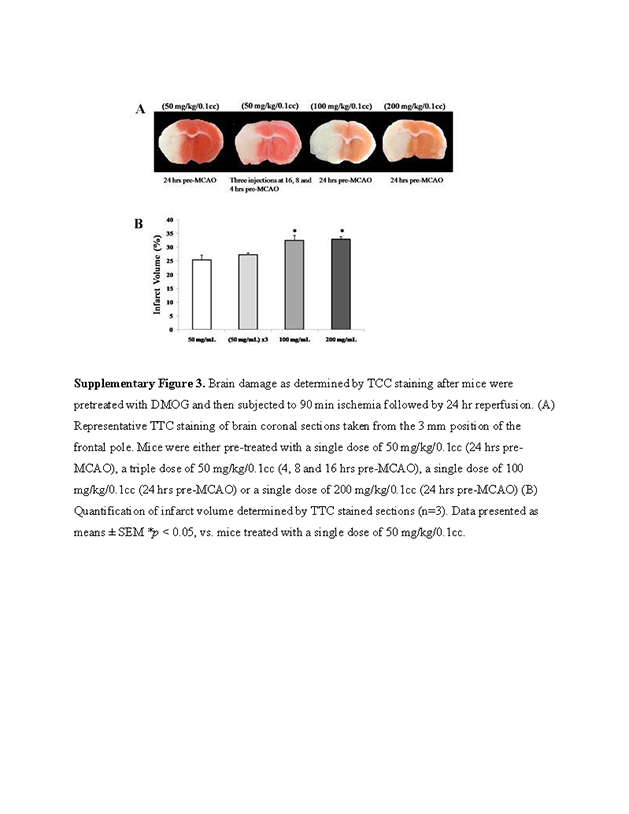

Supplement: Supplementary file 3 [file Image3.TIF]
